# Dual mode of action of metformin on mitochondrial metabolism

**DOI:** 10.1186/2049-3002-2-S1-P4

**Published:** 2014-05-28

**Authors:** Sylvia Andrzejewski, Simon-Pierre Gravel, Julie St-Pierre, Michael N Pollak

**Affiliations:** 1 Department of Biochemistry and Goodman Cancer Research Centre, McGill University, Montreal, Quebec, Canada; 2 Lady Davis Research Institute, Jewish General Hospital, Montreal, Quebec, Canada

## 

Metformin is commonly used in the treatment of diabetes, however recently there is increasing interest in “repurposing” the drug for cancer prevention or treatment. Metformin is believed to act by inhibiting mitochondrial complex I, leading to activation of AMPK. Interestingly, metformin treatment is not associated with the serious health consequences that are seen with classic inhibitors of complex I. To address this apparent paradox, we investigated the impact of metformin on cellular bioenergetics using in-depth respirometry analyses coupled with stable isotope tracer experiments in both cells and isolated mitochondria. We show that cells treated with metformin display reduced respiration. Metformin reduces respiration by specifically inhibiting respiration coupled to ATP production, while increasing uncoupled respiration. Thus, cells treated with metformin devote a large fraction of their respiration for uncoupled reactions and become inefficient. The impact of metformin on cellular respiration can be attributed to direct action on mitochondria as metformin inhibits complex I-dependent respiration and citric acid cycle activity in isolated mitochondria. Overall, our study reveals global bioenergetic consequences of metformin exposure, and establishes mechanistic similarities between metformin and recently described liver-specific uncoupling agents that, like metformin, improve the metabolic consequences of obesity.

